# Respiratory Tract Reinfections by the New Human Metapneumovirus in an Immunocompromised Child

**DOI:** 10.3201/eid0809.020238

**Published:** 2002-09

**Authors:** Gilles Pelletier, Pierre Déry, Yacine Abed, Guy Boivin

**Affiliations:** * Centre Hospitalier Universitaire de Québec and Laval University, Québec City, Québec, Canada

**Keywords:** human metapneumovirus, paramyxovirus, respiratory tract infection, immunosuppression

## Abstract

The human Metapneumovirus (HMPV), a new member of the Paramyxoviridae family, has been recently associated with respiratory tract infections in young children. We report the case of a young, immunocompromised child who had severe lower respiratory tract infections during two consecutive winter seasons caused by genetically distinct HMPV strains.

A variety of viruses,—such as the influenza viruses A and B, the Human respiratory syncytial virus (HRSV), the parainfluenza viruses, and the adenoviruses—cause seasonal respiratory tract infections in young children. Symptoms range from influenzalike illnesses to lower respiratory tract syndromes, such as bronchiolitis, croup, and pneumonitis (1–4). These viruses may also cause severe respiratory infections in immunocompromised patients (1,4). However, the etiology of many cases of bronchiolitis and pneumonitis remains unknown despite the extensive use of sensitive diagnostic techniques.

The human metapneumovirus (HMPV) has been recently classified as a new member of the Paramyxoviridae family based on nucleic acid sequence, gene organization, and electron microscopy findings (5–7). This virus has been reported to cause respiratory tract infections in children <5 years of age from the Netherlands (5), as well as in elderly patients from North America (7). We describe a case of recurrent HMPV respiratory tract infections, associated with genetically distinct viral strains, in an immunocompromised child.

## Case Report

A 7-month-old girl, who had acute lymphoblastic leukemia with meningeal involvement (treated with intravenous vincristine and doxorubicin, as well as intrathecal methotrexate), was brought to the hospital on March 23, 1998, for recent onset of nasal congestion and nonproductive cough. Her medical history showed that she had recent contact with family members who had a common cold. On initial physical examination, her temperature was 38.9°C with a respiratory rate of 40 per min, with no rales at lung auscultation. Initial laboratory tests showed a leukocyte count of 1,800 X 109 cells/L with 25% neutrophils and 3% band forms. The child was hospitalized and treated empirically with intravenous ceftazidime. Urine and blood cultures were negative for bacterial pathogens. The patient rapidly improved and was discharged from the hospital on day 3 after admission; no antibiotics were given, and the presumptive diagnosis was viral upper respiratory tract infection. The next day, the patient was brought to the same hospital for an exacerbation of coughing and sneezing. On physical examination, she had a temperature of 38.5°C, with bilateral wheezing at lung auscultation. The respiratory rate was 36 per min, and a clear nasal discharge was noted. The leukocyte count was 1,600 x 109 cells/L, with 33% neutrophils and 0% band forms. Blood was drawn for cultures, and a nasopharyngeal aspirate (NPA) sample was obtained for HRSV antigenic testing (Test Pack, Abbott Laboratories, Abbott Park, IL) and viral culture on Madin Dorby Kidney cells (MDKC), tertiary monkey kidney cells (LLC-MK2), Hep-2, human foreskin fibroblast, Vero, Mink lung, human lung adenocarcinoma (A-549), human rhabdomyosarcoma (RD), transformed human kidney 293, and human colon adenocarcinoma (HT-29) cells. The patient was again treated empirically with intravenous ceftazidime. Three days after admission her condition had improved, and she was discharged from the hospital. The final diagnosis was bronchiolitis, and antibiotics were not given. Bacterial blood cultures and the rapid antigenic test for HRSV were negative. However, after 17 days of incubation, the viral culture from the NPA showed a nonhemagglutinating virus (isolate 1) growing in LLC-MK2 cells. Immunofluorescence assays with antibodies against common respiratory pathogens (influenza viruses A and B, HRSV, adenoviruses, and parainfluenza viruses 1–4) were all negative.

The next year, on January 18, 1999, during a scheduled medical appointment for administration of intravenous (daunorubicin and vincristine) and intrathecal (methotrexate, cytarabine, and hydroxyvrea) chemotherapy in addition to oral dexamethasone, the now 17-month-old girl again had nasal congestion and nonproductive cough. Her father mentioned that he had an upper respiratory tract infection 2 weeks earlier. An NPA sample was obtained for viral culture and HRSV antigenic detection. The patient did not appear ill and was allowed to go home the same day after chemotherapy. The HRSV antigenic test came back negative, but the viral culture showed an unidentified cytopathic effect only apparent in LLC-MK2 cells after 14 days of incubation (isolate 2). Immunofluorescence assays were again negative for all common respiratory viruses. The patient received another course of intravenous chemotherapy (daunorubicin and vincristine) on January 25 at the outpatient clinic, and her upper respiratory tract symptoms were then treated with an oral antibiotic (axetil cefuroxime).

Five days later, on January 30, 1999, the child was admitted to the hospital with a fever (39.9°C), persistent dry cough, and clear nasal discharge while on oral antibiotic. On physical examination, her respiratory rate was 28 per min with fine bilateral crackles at lung auscultation. An otoscopic exam showed bilateral otitis. The leukocyte count showed neutropenia (0.400 x 109 cells/L), and ceftazidime plus vancomycin were initiated. The blood cultures taken on the first day of hospitalization indicated a coagulase-negative staphylococcus (1 of 2 bottles were positive). On the fourth day of hospitalization, the patient’s clinical condition deteriorated with desaturation (pO2, 0.88) and tachypnea (respiratory rate, 60–70 per min). A chest x-ray showed bilateral infiltrates compatible with pneumonitis. The next day, an NPA sample was obtained for a viral culture and direct immunofluorescent assays against typical respiratory viruses. The results were negative. Intravenous erythromycin was administered for empirical coverage of atypical pathogens. On day 10 of hospitalization, the patient was transferred to the pediatric intensive-care unit and was intubated. A bronchoscopy was performed, and a bronchoalveolar lavage (BAL) sample was obtained for cultures of fungal, mycobacterial, viral, and bacterial pathogens, as well as for specific staining procedures for Pneumocystis carinii, fungi, bacteria, and mycobacteria. At that point, ceftazidime was replaced by trimethoprim/sulfamethoxazole, and an amphotericin B lipidic complex formulation (Abelcet, Liposome, Canada) was added. All cultures and specific staining procedures performed with the BAL sample were negative. Direct immunofluorescence assays on the BAL sample also were negative for influenza A and B, parainfluenza 1–4, and HRSV. On day 13 of hospitalization, the patient’s respiratory signs deteriorated markedly, with bilateral pulmonary infiltrates compatible with acute respiratory distress syndrome. Four days later, because of free air in the pericardium and peritoneum (the patient was on high ventilatory pressure), drainage tubes were placed, resulting in a temporary improvement of pulmonary gas exchange. New blood cultures drawn on day 27 were negative. The next day, shock developed, and vasopressive drugs were started. Because of her irreversible condition, all treatment was stopped on day 30, and the patient died shortly thereafter. No autopsy was performed.

Two years after the child’s death, reverse transcription-polymerase chain reaction assays for HMPV with infected LLC-MK2 cell culture supernatants of the two unidentified isolates were performed and found to be positive. On the basis of partial published sequences, a set of primers was designed for amplification of the F gene of HMPV (5,7). The forward primer sequence was 5´-ATGTCTTGGAAAGTGGTG-3´, and the reverse primer sequence was 5´-TCTTCTTACCATTGCAC-3´. Amplified products were sequenced by an automated DNA sequencer (ABI 377A [Perkin-Elmer Applied Biosystems, Foster City, CA]) and the 759-bp nucleotide sequences of the two viral isolates from consecutive seasons were aligned with the Clustal W software (8). The two viral isolates differed by 114 nt in the F gene sequence, which resulted in a change of 10 amino acids (Figure).

**Figure:**
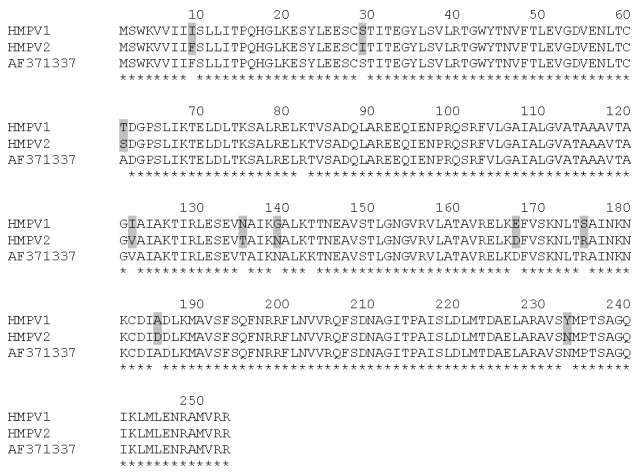
Comparison of the partial amino acid sequences of Human Metapneumovirus (HMPV) isolates 1 (recovered in 1998) and 2 (recovered in 1999) for the fusion protein (residues 1 to 253). The sequences were aligned with the reference sequence from the Netherlands (GenBank accession no. AF371337). Asterisks denote identical residues; the shaded boxes highlight different amino acids between the two HMPV isolates from this study.

## Conclusions

We describe the case of a young immunocompromised child who had respiratory tract infections during two consecutive seasons, probably due to HMPV, a newly discovered paramyxovirus. On the basis of sequence analysis of the viral F gene, we found that the child was infected by two genetically distinct HMPV strains.

In the first clinical episode (occurring at the age of 7 months), the initial symptoms resembled a common cold and progressed to bronchiolitis, followed by a complete resolution of symptoms within a few days. HMPV was the only organism isolated in culture from an NPA sample, and the HRSV rapid antigenic test was negative. Ten months later, the child had a second respiratory tract infection, again with common cold symptoms. HMPV was again the sole pathogen isolated in an NPA sample. In the next weeks, however, the patient’s clinical condition deteriorated, with development of bilateral pneumonitis and respiratory failure and ultimately death. The BAL sample obtained showed no bacterial, viral, or fungal pathogens by culture, antigenic testing, or special staining procedures. The exact cause of respiratory failure in this immunocompromised child remains uncertain, because autopsy was not performed. An opportunistic infection or a neoplastic infiltrate was the most probable cause. HMPV could have led to the acute respiratory distress syndrome in this immunocompromised patient. We suggest that the initial upper respiratory tract infection may have progressed to pneumonitis after the patient received intensive chemotherapy. The fact that HMPV was not recovered in the last BAL sample does not rule out such viral infection since paramyxoviruses are very labile (1), and the culture may not have been incubated long enough for a cytopathic effect to be observed.

The two HMPV isolates from this study had many differences at the nucleotide level (similarity 85.0%) but much less at the amino acid level (similarity 96.0%) in the gene coding for the fusion (F) protein. Isolate 2, recovered in 1999, had greater amino acid similarity with the prototype sequence from the Netherlands (5) compared with isolate 1 (98.0% vs. 96.0% similarity). Furthermore, the two strains seem to belong to the two different HMPV lineages previously reported by Dutch and North American groups (5,7). We suggest that many HMPV strains may cocirculate in a specific population, and such viral diversity, coupled with waning immunity, as found in elderly and immunocompromised patients, may lead to multiple reinfections similar to HRSV (2,9–12).

Future studies are needed to evaluate the full clinical spectrum associated with HMPV infection, the groups at risk for severe complications, and the potential therapeutic options. Our data suggest that HMPV should be added to the list of pathogens associated with severe respiratory tract infections in immunocompromised patients.
